# The Buffalo Provident Dispensary and Hospital Association

**Published:** 1883-03

**Authors:** 


### The Buffalo Provident Dispensary and Hospital Association.

The high character of the gentlemen who constitute the consulting and attending staff of this association gives assurance of the organization of an agency long needed in meeting a class of patients which, for several years, have been deprived of the benefit of professional assistance.

As implied in the name selected for the Dispensary, it is not intended to pauperize the public by giving with too lavish a hand professional services to all who apply. It is the aim to discriminate, as far as possible, and to co-operate with the various charities now existing in the city, to extend to the worthy poor the best medical aid it is possible to render. With such motives the Provident Dispensary cannot fail to be successful, and we trust that the principles on which it is based will be faithfully carried out in the administration of its interests. We give the names of its officers and the consulting and attending staff:

OFFICERS.

President—William S. Tremaine, M. D.
Vice-President—A. R. Davidson, M. D.
Treasurer—Dougal Macniel, M. D.
Secretary—Henry D. Ingraham, M. D.

CONSULTING STAFF.

Surgeons—Charles C. F. Gay, M. D.; John Cronyn, M. D.; John Boardman, M. D.

Physicians—John Hauenstein, M. D.; Edward Tobie, M. D.; George N. Burwell, M. D.

ATTENDING STAFF.

Surgery—William S. Tremaine, M. D.; Herman Mynter, M. D.

Diseases of Women—Thomas Lothrop, M. D.; Henry D. Ingraham, M. D.; Carlton C. Frederick, M. D.

Diseases of the Digestive Organs and Kidneys—A. R. Davidson, M. D.

Diseases of the Throat and Chest—Charles G. Stockton, M. D.

Diseases of Children and the Nervous System—Joseph W. Keene, M. D.; Frederick Peterson, M. D.

Skin Diseases—Dougal Macniel, M. D.

Diseases of the Eye and Ear—Frank W. Abbott, M. D.

The Dispensary has opened its rooms at 385 Washington street, and provides excellent facilities for the work it aims to perform.

